# Supplementary material for: Chromothripsis during telomere crisis is independent of NHEJ, and consistent with a replicative origin
Source: Genome Res. 2019 May;29(5):737–49. doi: 10.1101/gr.240705.118 (PMC6499312; doi:10.1101/gr.240705.118)
Supplement: Supplemental Material [file supp_gr.240705.118_Supplemental_file_1.zip › contigs/annotated_contigs/DB113/contig.2.DB113_length_621_mean_cov_5.40901771337.docx]

**DB113_length_621_mean_cov_5.40901771337**

```
ACTGCTATCTAATTCTAGAACATTTTGTCACTCCAAAAGAAACCCTATACCCACTCCCCATTCATGTCCAGCACCTGGGAATTCCTAAT
 >chr10:12934939-12935231 + E=6e-155
CTACTCTCTGTCCCTTTCTTTTTCTCCTCTCCTTCTGGGACTCTCAATATGTGTATGTTGGTCCACTTGATAGCATCCTGTACATCTCT

TAGGCTCCGTTCATTTTTCTTCATTCCTTTTTATATCTCTTTCTCAGCCTGGATAATCTATAAGTTCACTGATTATTCTGCTTGTTCAA

ATCTGCTGTTGAGCCCCTCTA|GTGA|CATCCTCCATACCCTAAGTCCACTTGTGCCTTTTTAATTCTATTTGTTTCAGCCACAAACTT
                      >chr10:13279013-13279303 + E=2e-131
GTATTGAACACCTGCTGCCATGCACCAGGCACCTGTGCCCGTCACTGGCGAGTCAGCCTTGGGTAAATCATTTTTAAAATTTTTTCAGC

TGGGCGTGGTGGCGCATGCCTATAATCTCAGCACTTTGGGAGGCCGAGGCAGGCAGATCACGAGGTCAGCAGTTCGAGACCAGCCTGGC

CAACATGGTGAAACCCCGTCTCTACTAAAAATACAAAAAATTAGC|CGGGCCTGGTGGCACGCGCCTGTAATCCCAGCTACTCGGGTGG

C
```
